# Abnormal Uterine Bleeding among Women Visiting Gynecology Out-patient Department of a Tertiary Care Hospital: A Descriptive Cross-sectional Study

**DOI:** 10.31729/jnma.6954

**Published:** 2022-02-28

**Authors:** Deepak Shrestha, Shreyashi Aryal, Archana Tiwari, Rupesh Sharma

**Affiliations:** 1 Department of Obstetrics and Gynecology, Lumbini Medical College and Teaching Hospital, Paipa, Nepal; 2 Department of Pathology, Lumbini Medical College and Teaching Hospital, Palpa, Nepal; 3 Department of Radiodiagnosis and Imaging, Lumbini Medical College and Teaching Hospital, Palpa, Nepal

**Keywords:** *biopsy*, *endometrial hyperplasia*, *postmenopausal women*, *uterine bleeding*

## Abstract

**Introduction:**

Abnormal uterine bleeding accounts for more than 70% of complaints in peri- and post-menopausal women. The main purpose of investigating them is to rule out premalignant and malignant endometrial lesions. This study aimed to find out the prevalence of abnormal uterine bleeding among out-patients visiting the gynecology department of a tertiary care hospital.

**Methods:**

This descriptive cross-sectional study was conducted in the Department of Obstetrics and Gynecology of a tertiary care center from August 1, 2020 to April 23, 2021. Ethical approval was obtained from the Institutional Review Committee of the same institution (IRC-LMC 07-C/020). A total of 2680 women visiting gynecology outpatient departments were included by convenience sampling technique. Women with abnormal uterine bleeding were evaluated by endometrial thickness measurement and endometrial biopsies. Statistical Package for the Social Sciences version 16 was used for data analysis. Point estimate at 95% Confidence Interval was calculated along with frequency, percentage, mean and standard deviation.

**Results:**

Among 2680 women visiting the gynecology outpatient department, the prevalence of abnormal uterine bleeding was 240 (8.9%) (7.82-9.98 at 95% Confidence Interval). The mean endometrial thickness was 10.15±6.63mm. Majority of patients 104 (43.3%) had endometrial thickness >10mm. Sixty-six (27.8%) patients had disordered proliferative endometrium while endometrial carcinoma was noted in three (1.2%) patients. Atrophic endometrium was observed in 17 (7.2%) and endometrial hyperplasia in nine (3.8%) patients.

**Conclusions:**

The prevalence of abnormal uterine bleeding was found to be higher in comparison to other studies. Disordered proliferative endometrium was the most common histopathological finding followed by secretory phase endometrium.

## INTRODUCTION

Abnormal uterine bleeding (AUB) is a very common presentation in the peri- and post-menopausal women, accounting more than 70% of their complaints.^1^ It may be defined as the bleeding pattern that differs in frequency, duration, and amount from a normal menstrual pattern.^2^ The main aim of investigating these women is to rule out endometrial cancer and its precursor lesions.^3^

Vaginal or cervical cytologic studies do not serve as sufficiently accurate screening methods for the detection of endometrial carcinoma, and direct intrauterine cell sampling and hysteroscopy are not always practical because of their invasive nature and need for expertise.^4^ Transvaginal sonography (TVS) is an acceptable and non-invasive modality with good clinical applicability for the evaluation of endometrial thickness, and it shows a good correlation with pathology results.^5^

This study aimed to find out the prevalence of abnormal uterine bleeding among out-patients visiting gynecology department of a tertiary care hospital.

## METHODS

This was a descriptive cross-sectional study conducted in the Out-patient Department (OPD) of the Department of Obstetrics and Gynecology of Lumbini Medical College and Teaching Hospital of a tertiary care hospital (LMCTH), Palpa, Nepal. The data were collected over a period of nine months from August 1, 2020 to April 23, 2021. Ethical approval was obtained from the Institutional Review Committee (IRC-LMC07-C/020) prior to data collection. Informed consent were obtained from individual patients. The confidentiality of the patients was maintained throughout the study and information regarding the identification of the patients was recorded anonymously. Convenience sampling technique was employed.

The sample size for the study was calculated employing the given formula,

n = Z^2^ × p × q / e^2^

  = (1.96)^2^ × 0.5 × (1-0.5) / 0.02^2^

  = 2401

Where,

n = minimum required sample size
Z = 1.96 at 95% Confidence Interval (CI)
p = prevalence taken as 50% for maximum sample size
q = 1-p
e = margin of error, 2%

We added 10% of the sample size to curb for nonresponse rate thus final sample size of 2668 was calculated. However, we took a total of 2680 patients who visited the gynecology OPD during the study period. Medical history was obtained and the patients underwent general physical and gynecological examinations. Those with a history of bleeding disorders, adnexal masses, premalignant or malignant lesions of the vulva/vagina, and those receiving hormone replacement therapy were excluded.

Abnormal Uterine Bleeding (AUB) was clinically diagnosed when women were presented with bleeding pattern that differs in frequency, duration and amount from a pattern observed during a normal menstrual cycle or after menopause.^6^ Chronic AUB is defined as abnormal uterine bleeding for at least four out of six months while acute AUB is termed as a single episode of severe uterine bleeding that is sufficient to require immediate intervention to prevent further blood loss.^7^

All the patients clinically diagnosed with AUB underwent TVS examination. Endometrial thickness was measured at the thickest part of the endometrium in the longitudinal plane by a 7.5MHz vaginal transducer. Both endometrial layers from one basalis to the contralateral basalis through the uterine cavity were included. The Endometrial Thickness was then noted in millimeters into the report. The ultrasonogram (USG) examination was done by the on-duty radiologist on the given day as subjecting all the patients to a single radiologist was not feasible due to logistics reasons. Endometrial biopsy was taken in the OPD procedure room for outpatients. After informed consent, the patients were given an oral analgesic (a combination of ibuprofen and paracetamol) half an hour prior to the procedure. The biopsy was taken using a Manual Vacuum Aspiration (MVA) syringe and IPAS® cannula. The sample thus collected was transferred into a container with 10% formalin and sent for histopathological examination (HPE) to the pathology laboratory.

The endometrial samples were reviewed by a single expert pathologist for the diagnosis. The endometrial tissues were fixed in 10% formalin and processed. The paraffin-embedded tissues were sectioned at 3-4pm followed by hematoxylin and eosin staining. Thus, obtained sections were studied by the pathologist under light microscopy. The HPE reports were categorized into seven categories: atrophic/inconclusive endometrium, endometritis, normal endometrium, hormonal changes, disordered proliferative endometrium, endometrial hyperplasia, and endometrial carcinoma.

The data were entered, coded, and analyzed with Statistical Package for the Social Sciences (SPSS) version 16. Point estimate at 95% Confidence Interval was calculated along with frequency, percentage, mean and standard deviation.

## RESULTS

Of the total 2680 patients attending the gynecology OPD, the prevalence of AUB was 240 (8.9%) (7.829.98 at 95% Confidence Interval). In three of them, endometrial thickness was not recorded thereby allowing 237 cases for further evaluation. Thirty-three (13.7%) patients were postmenopausal while 207 (86.3%) patients presented with AUB in pre-or peri-menopausal period. The mean age of the patients was 45.04±7.93 years ranging from 19 to 72 years.

**Figure 1:**
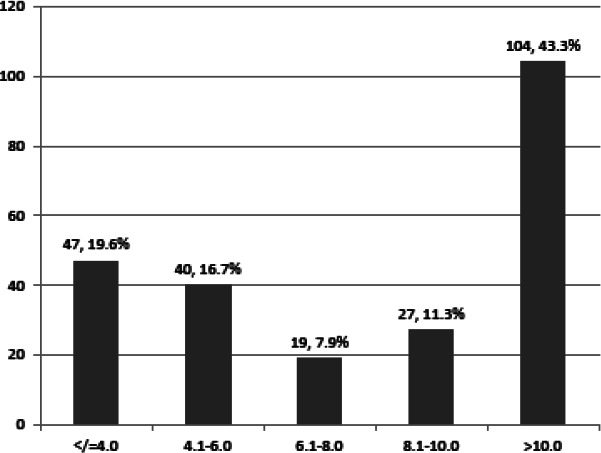
Distribution of cases according to endometrial thickness (n= 237).

The mean endometrial thickness as measured by TVS was 10.15±6.63mm with a range of 1.90 to 36.30mm. For premenopausal and postmenopausal women, the mean endometrial evaluation were 10.66±6.74mm and 7.01 ±4.88mm respectively. The majority of patients 104 (43.3%) had endometrial thickness >10mm. Endometrial thickness of ≤4mm was found in 47 (19.6%) patients (Figure 1).

We found that 66 (27.8%) patients had disordered proliferative endometrium (DPE) while endometrial carcinoma was noted in three (1.2%) patients. Two of them had endometrioid adenocarcinoma and one had endometrial stromal neoplasm. All three of them underwent surgical management. Atrophic endometrium was observed in 17 (7.2%) and endometrial hyperplasia was reported in nine (3.8%) patients (Table 1). In this study, the benign endometrial conditions were found in 225 (94.93%) and that of endometrial hyperplasia and malignancy was 12 (5.06%).

**Table 1:** Frequencies of the histopathological diagnoses (n = 237).

**Histopathology**		n (%)
**Atrophic endometrium**		17 (7.2)
**Inconclusive**		4 (1.7)
**Endometritis**	Acute endometritis	3 (1.3)
	Chronic endometritis	24 (10.1)
	Acute on chronic endometritis	4 (1.7)
**Normal endometrium**	Proliferative phase endometrium	39 (16.5)
	Secretory phase endometrium	57 (24.1)
	Benign endometrial tissue	6 (2.5)
**Hormonal changes**		5 (2.1)
**Disordered proliferative endometrium**		66 (27.8)
Endometrial hyperplasia	Without atypia	7 (3)
	With atypia	2 (0.8)
**Endometrial carcinoma**	Endometrioid adenocarcinoma	2 (0.8)
	Endometrial stromal neoplasm	1 (0.4)

In normal endometrium, which included proliferative and secretory phase endometrium too, 17 cases had endometrial evaluation ≤4mm and 43 cases had endometrial evaluation >10mm (Table 2). Most of them were secretory phase endometrium 57 (24.1%) meaning they were mostly taken in the secretory phase of the cycle. In atrophic endometrium, five cases had endometrial evaluation >10mm while 12 cases had endometrial evaluation ≤4mm. Out of nine cases of endometrial hyperplasia, six cases had endometrial evaluation >10mm while one case was missed at a cutoff of 4mm. Only one case with endometrial hyperplasia was postmenopausal while two out of three women diagnosed with malignancy were postmenopausal.

**Table 2:** Comparison of endometrial thickness with histopathology (n = 237).

Histopathology	Endometrial thickness range (mm) n (%)					Total n (%)
	≤4.0	4.1-6.0	6.1-8.0	8.1-10	>10.0	
Endometritis	9 (3.7)	9 (3.7)	1 (0.4)	1 (0.4)	11 (4.6)	31 (13.0)
Normal	17 (7.1)	17 (7.1)	10 (4.2)	15 (6.3)	43 (18.1)	102 (43.0)
Atrophic/inconclusive	12 (5.0)	3 (1.2)	1 (0.4)	-	5(2.1)	21 (8.8)
Disordered proliferative	8 (3.3)	9 (3.7)	6 (2.5)	6 (2.5)	37 (15.6)	66 (27.8)
Hormonal changes	-	1 (0.4)	-	3 (1.2)	1 (0.4)	5 (2.1)
Endometrial hyperplasia	1 (0.4)	-	-	2 (0.8)	6(2.5)	9 (3.7)
Endometrial malignancy	-	1 (0.4)	1 (0.4)	-	1 (0.4)	3(1.2)

## DISCUSSION

Abnormal uterine bleeding is one of the common diagnoses in gynecology OPD and ultrasonogram (USG) is always the first line of investigation. Apart from looking for the obvious structural lesions or abnormalities of the uterus, measurement of endometrial thickness remains a key objective of this USG. The present study aimed to evaluate the prevalence of AUB and examine the histopathological pattern of endometrial biopsies in patients presenting with AUB. The prevalence of AUB was 8.9% in the present study. This finding is much higher compared to the prevalence of 4.7% reported by Sedhain L, et al.^8^ The relatively lower prevalence in their study might be due to the inclusion of both gynecologic and obstetric patients in the total sample population. Another study by Chapagain S, et al. also found a lower prevalence of 4.8% in their study.^9^

The mean age of our study population was 45.04±7.93 years. The study showed that the incidence of AUB is more common in the fifth decade of life. This finding is in agreement with other studies too.^10-12^ Thirty-three (13.7%) women were postmenopausal which is comparable to the study by Sur D, et al. (10.43%) and Tiwari A, et al. (17%).^3,11^ The proportion of benign endometrial conditions, premalignant and malignant endometrial conditions in our study were found to be 94.93%, 3.79%, and 1.27% respectively. However, in a prospective study by Kumari A, et al, the incidence of benign condition was 49%, premalignant 9%, and malignant 42%, respectively.^10^ This contradictory finding is because the study was done in a gyne-oncological setup dealing more with premalignant and malignant cases.

In this study, a substantial percentage (13.1%) of the samples was reported as having endometritis which is similar to a previous study (15%) done in the same setting in a smaller sample size.^11^ However, another study has reported only 3.06% as endometritis.^3^ This difference is probably because women in our region suffer more from infections owing to a number of reasons as poor hygiene, poor socioeconomic status, and accessibility to health care. Also, proliferative and secretory phase endometrium were seen only in 16.5% and 24.1% cases in our study as compared to 32.74% and 26.38% in the study by Sur D and Chakravorty R.^3^ This might be because disordered proliferative endometrium was not categorized as a separate entity in the study as in ours. In fact, disordered proliferative endometrium 66 (27.8%) constituted the most common histological pattern in our study. Another study reported the proportion of proliferative, secretory, and hyperplastic endometrium as 37%, 16%, and 31% respectively.^13^ This difference could be because of the difference in sample size of the study.

Compared to a previous study,^11^ the prevalence of disordered proliferative endometrium found in this study is more and that of proliferative endometrium is less. This might be attributed to the shifting magnitude of the disease pattern. Similarly, the study by Vaidya S, et al. reported the incidence of proliferative, secretory and disordered proliferative endometrium as 24.10%, 29.64% and 17.59% respectively.^12^ Endometrial hyperplasia is generally taken above a cut-off of ≥4mm,^10^ or ≥5mm,^13^ for postmenopausal women, and 11mm for premenopausal women.^10^ In another study, the cut-off values were taken at ≥8mm in the proliferative phase and ≥16mm in the secretory phase for premenopausal women.^14^ However, only seven cases with endometrial evaluation >10mm in our study were found to have endometrial hyperplasia or malignancy. In fact, most of the patients with endometrial evaluation >10mm had either normal endometrial or disordered proliferative patterns. The Nordic multicentric trial reported a sensitivity of 96% and specificity of 68% to detect endometrial abnormalities in postmenopausal women at a cut-off value of endometrial evaluation of 4mm.^15^ But there is a lack of clearly defined cut-off value for endometrial hyperplasia in premenopausal women.^3^

In this study, 43 cases with normal endometrium had endometrial evaluation >10mm. Most of them were secretory phase endometrium 57 (24.1%) meaning they must have been taken in the secretory phase of the cycle. Interestingly, five cases with atrophic/inconclusive histopathological patterns had endometrial evaluation >10mm. Women with atrophic endometrium can have thickened endometrium on TVS because the cavity might be distended by polyp or fluid which is often difficult to remove by blind curettage. One case in the study with endometrial hyperplasia had endometrial evaluation <4mm. But this case was not missed because all the cases presenting with AUB in peri- and post-menopausal age group were subjected to endometrial biopsy. In the study by Singh P, et al., among patients with normal and atrophic endometrium, the majority had endometrial evaluation <4mm and those with hyperplasia had endometrial evaluation >4mm and with polyp had endometrial evaluation >6mm.^16^

In our study, out of 66 cases of disordered proliferative endometrium 37 had endometrial evaluation >10mm. This shows that women with disordered proliferative endometrium were more likely to have thickened endometrium. Our study found that 43.3% of patients had endometrial evaluation >10mm and 19.6% had endometrial evaluation ≥4mm. This finding that more than 60% of the study population had endometrial evaluation either ≥4mm or >10mm suggests that those having endometrial evaluation within this range are the ones more likely to have abnormal bleeding. In this study, 43 (42.16%) out of 102 patients with normally reported endometrial biopsies had endometrial evaluation >10mm. Most of these biopsies might have been taken in the secretory phase of the menstrual cycle. In fact, 57 patients had secretory phase endometrium in the study.

There are a few limitations of this study. It did not take into account the day of the menstrual cycle on which endometrial biopsy was taken. The inter-observer bias among the radiologists for the measurement of endometrial evaluation was not corrected.

## CONCLUSIONS

The prevalence of abnormal uterine bleeding was higher than other similar studies conducted in different parts of the country. After normal endometrium, disordered proliferative endometrium was the most common histopathological finding followed by secretory phase endometrium which reflects the changing pattern of endometrial pathology as compared to various other publications. Atypical endometrial hyperplasia and endometrial carcinoma constituted a small fraction of abnormal uterine bleeding similar to other studies.
